# A Meta‐Analysis of Multidimensional Cognitive Functions Changes in Different Intensities of High‐Altitude Hypoxia

**DOI:** 10.1002/brb3.70883

**Published:** 2025-09-21

**Authors:** Yan Jiang, Ke‐Er Cai, Ling‐Ling Zhu, Ming Fan, Yong‐Qi Zhao, Du‐Ming Wang

**Affiliations:** ^1^ Department of Psychology Zhejiang Sci‐Tech University Hangzhou China; ^2^ Beijing Institute of Basic Medical Sciences Beijing China; ^3^ School of Information Sciences & Engineering Lanzhou University Lanzhou China

**Keywords:** cognition function, high‐altitude, high‐altitude acclimation, hypoxia, meta‐analysis

## Abstract

**Purpose:**

This meta‐analysis aims to comprehensively evaluate the impact of high‐altitude hypoxia on multidimensional cognitive functions, considering moderating variables such as altitude, exposure time, cognitive domain, and experimental design.

**Methods:**

A three‐level meta‐analysis was conducted on 59 studies (1966–2024) comprising 739 effect sizes. Studies were screened and coded according to Preferred Reporting Items for Systematic Reviews and Meta‐Analyses (PRISMA) guidelines. Cognitive tasks were categorized into seven domains: executive control, working memory, long‐term memory, perceptual ability, psychomotor skill, attention, and others. Hedges’ *g* was used as the effect size measure, and multilevel modeling was applied to account for dependent effect sizes.

**Findings:**

High‐altitude hypoxia significantly impaired overall cognitive function (*g* = −0.424, *p* < 0.001). Long‐term memory and perceptual functions were most affected, followed by executive control, attention, and psychomotor skills; working memory was least impacted. Cognitive impairment was significant at altitudes above 2500 m. Acute (< 3 days) and chronic (> 30 days) exposure significantly affected cognition, while intermediate exposures (3–30 days) did not. Subgroup analyses revealed varying sensitivity across cognitive domains to altitude and exposure duration.

**Conclusion:**

High‐altitude hypoxia adversely affects cognitive function, with severity varying by cognitive domain, altitude, and exposure duration. These findings highlight the need for tailored interventions and further research into acclimatization and de‐acclimatization processes.

## Graphical abstract

High‐altitude hypoxia environment negatively affected overall cognitive function. It had a more pronounced impact on long‐term memory and perceptual functions, followed by executive control, attention, and psychomotor functions, with working memory being the least affected, and altitude (> 2500m) and exposure time (< 3 days and > 30 days) are significant moderators.

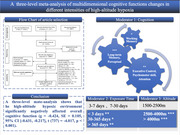

## 1 Background

The uplift of the Earth's crust across vast areas over hundreds of millions of years has created a terrain with varied relief. This region is also referred to as the “stage of the Earth” due to its expansive area, open topography, and prominent steep slopes.

Approximately 500 million people reside permanently in high‐altitude areas at elevations 1500 meters above sea level (Tremblay and Ainslie 2021). Increasing numbers of people are traveling to high altitudes for tourism, work, and study, with even high mountains above 5500 m bearing the footprints of thousands of hikers and climbers (Gallagher and Hackett 2004). Hypobaric hypoxia, the most prominent feature of the high‐altitude environment, has become a key focus of research in high‐altitude medicine. Hypoxia can impair various physiological systems, including the nervous system (Zhao et al. 2008). The physiological damage caused by high‐altitude hypoxia conditions affects the brain., which subsequently impacts cognitive function (Wilson et al. 2009). Consequently, cognitive changes resulting from hypoxia have become an increasingly significant public health concern (Tao et al. 2024).

The brain, the body's most oxygen‐dependent organ, requires over 20% of inhaled oxygen to sustain daily cognitive functions (Attwell et al. 2010; Raichle and Gusnard 2002). When individuals from sea‐level regions travel to the highlands, as altitude increases, the atmospheric pressure decreases, which reduces the partial pressure of oxygen (Gonggalanzi et al. 2016). Consequently, oxygen molecules become more dispersed, leading to reduced oxygen availability. This results in lower oxygenation levels in the blood and lungs (Virués‐Ortega et al. 2011). Acute hypoxia induces rapid decrease in blood oxygen levels within minutes. This reduction impairs the oxygen‐binding capacity of arterial erythrocytes (Shukitt‐Hale et al. 1994; Singh et al. 2004; Tu et al. 2010) and triggers three primary physiological responses: (1) increased cerebral bold flow (Muza et al. 2010), (2) decreased arterial oxygen saturation, and (3) sympathetic nervous system activation leads to increased heart rate and increased myocardial contractility, which increases cardiac output (Bártsch et al. 2007). In response to these environmental changes, the cardiovascular, respiratory, and central nervous systems undergo various acclimatization to maintain homeostasis (Travers et al. 2022). Cognitive function is also impacted. In high‐altitude environments, as altitude increases, hypobaric hypoxia intensifies, raising the risk of cognitive impairment (Algaze et al. 2020) and reducing the ability to perform mental and physical tasks (Beier and Oswald 2012).

Numerous studies have shown that individuals exposed to hypoxia experience changes in cognitive functions, including vision (Kobrick 1976), attention (Espinoza‐Navarro and Grau 2014), judgment, memory (Jung et al. 2020; McMorris et al. 2017), arithmetic (Hogan et al. 2010), emotional control (Fagenholz et al. 2007; Pavlicek et al. 2005), and other senior cognitive abilities such as complex judgment and cognitive flexibility (Chen et al. 2016), all of which show varying degrees of decline. Neuroimaging studies also demonstrate the impact of hypoxia on cognitive function. After 30 days of exposure to a high‐altitude environment, healthy subjects showed significant increases in gray matter volume and cortical surface thickness in regions such as the precentral gyrus, postcentral gyrus, lateral occipital cortex, and temporal lobe (C. Fan et al. 2016). Prolonged exposure to hypoxia results in a reduction of gray matter volume in areas such as the bilateral prefrontal lobes, right cingulate gyrus, and left anterior central gyrus (Yan et al. 2011a). This impacts cognitive functions, including perception, attention (J. Zhang et al. 2010), memory (Yan et al. 2011b), and social adaptation (Virues‐Ortega et al. 2006).

However, some studies suggest that high‐altitude hypoxia exposure does not negatively affect cognition. For example, Richardson et al. (2011) found that high‐altitude hypoxia exposure did not impact neurophysiological function. Similarly, Ma. et al. (2015) indicates that there were no significant changes in reaction time or attentional accuracy during prolonged high‐altitude hypoxia exposure. Davies et al. (2011) even reported enhanced attentional function in individuals exposed to high‐altitude environments. Similarly, a neuroimaging study by Luo et al. (2023) showed no significant changes in brain structure after prolonged exposure.

From a researcher's perspective on experimental design, the variability between studies and the management of confounding factors can significantly affect the results. For instance, factors such as the characteristics of the subject group (immigrants vs. native residents), the type of experimental design used (within‐study or between‐study), the nature of the experimental conditions (field studies or laboratory studies), the cognitive tests employed, and the methods of cognitive classification must all be taken into account. These elements introduce additional influences that should be considered when examining the impact of high‐altitude hypoxia exposure on cognitive function.

First, altitude serves as an important factor in this study. As altitude increases, the availability of oxygen decreases, which affects cognitive performance. Research has demonstrated that reaction times can increase even at relatively low altitudes, such as 1500 m. Furthermore, other studies indicate a sustained decline in various cognitive functions above 2500 m (Virués‐Ortega et al. 2011). However, the altitude threshold for cognitive impairment may be higher for individuals who have prolonged exposure, potentially exceeding 4000 m.

Second, the duration of exposure is crucial to the outcomes observed. When individuals from low‐altitude regions enter a high‐altitude environment, they undergo three phases: acute exposure, subacute exposure, and chronic exposure (Yan 2014; Zubieta‐Calleja et al. 2007). These phases lead to changes in brain function due to physiological acclimatization, which in turn affect cognitive performance.

Experimental tasks that target different cognitive domains can influence the results of studies. It remains unclear whether high‐altitude hypoxia exposure affects all cognitive tasks uniformly or selectively impacts specific cognitive functions. In acute hypoxic conditions, previous studies have primarily compared central executive tasks with nonexecutive tasks, finding no significant differences between the two (McMorris et al. 2017; Taylor et al. 2016). However, more complex tasks that require higher levels of cognitive function appear to be more significantly affected by prolonged exposure to high altitude environment (Hill et al. 2014; Hornbein 2001).

The current study aimed to quantitatively assess the effects of high‐altitude hypoxia exposure on the overall cognitive functioning of individuals who have experienced a high‐altitude sojourn. Using meta‐analytic methods, the study also sought to explore changes in various aspects of cognitive functioning under different levels of hypoxia exposure. Additionally, it compared individuals at hiFgh altitudes to those from lowland areas, considering factors such as altitude, duration of exposure, type of cognitive tasks, and experimental design as potential moderating variables.

## 2 Methods

### 2.1 Literature Search and Screening

This study followed the Preferred Reporting Items for Systematic Reviews and Meta‐Analyses (PRISMA) guidelines and performed a systematic literature review of previous research to investigate the impact of hypobaric hypoxic environments at varying intensities on cognitive functions.

To ensure comprehensive coverage, a systematic review (Martin et al. 2019) was conducted, searching both Chinese and English literature up to December 2024. The databases utilized included China Knowledge, Web of Science, and PubMed. Articles were identified using keywords such as “memory,” “reaction time,” “cognition,” “executive function,” “attention,” “perception,” “processing speed,” and “neuropsychology,” combined with terms like “altitude,” “hypoxia,” “mountain,” and “high‐altitude.” These keyword combinations were employed to locate relevant studies across multiple disciplines.

**FIGURE 1:**
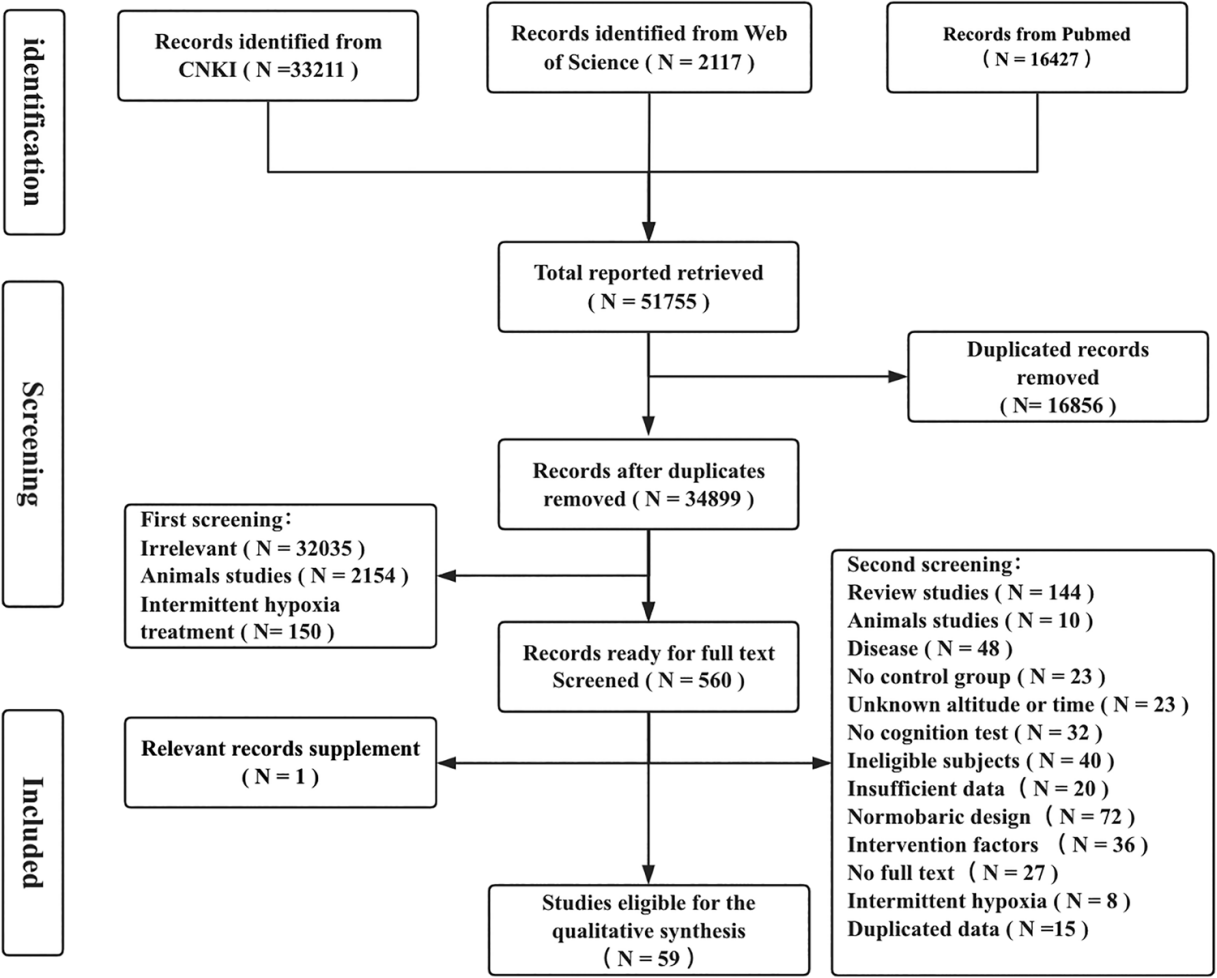
Flow chart of article selection.

All documents retrieved were managed using Microsoft Excel 2021 and EndNote 21.1 software. The first screening phase involved filtering based on the relevance of article titles, eliminating duplicates and articles clearly unrelated to the study topic. The remaining articles were further reviewed by examining their references to ensure a comprehensive literature search. The search process and the reasons for exclusion are presented in Figure 1.

### 2.2 Inclusion and Exclusion Criteria

The inclusion and exclusion criteria for this study were determined based on the PICOS (Patients, Interventions, Controls, Outcomes, and Study Design) principles.

The inclusion criteria for this study were as follows: (1) published articles in either Chinese or English, (2) the study population consisted of healthy individuals who were free from any physical or mental illnesses and were exposed to low‐pressure, low‐oxygen environments at altitudes of 1500 m (or 4291 ft) or higher, (3) cognitive function was assessed using appropriate experimental designs and testing instruments, and (4) the study design was either a within‐subjects or between‐subjects approach, with sea level serving as the control condition.

The exclusion criteria for this study were as follows: (1) studies involving pilots not acutely exposed to low‐pressure hypoxia, mountaineers with repeated exposure, minors, the elderly, and native highlanders (who were born and living in high altitude area); (2) studies that controlled for other variables (e.g., sleep deprivation, exercise, and medication) during low‐pressure hypoxia exposure; (3) studies lacking cognitive test data (e.g., number of subjects, mean, and standard deviation) or statistical comparisons between plains and high altitudes, and lacking post hoc test results in repeated measures experiments; (4) laboratory studies using hypoxic gas mixtures, oxygen masks, intermittent hypoxic treatments, or normobaric hypoxia; (5) studies that failed to specify the altitude and duration of low‐pressure hypoxic exposures; and (6) case reports, overviews, expert opinions, reviews, letters to the editor, animal studies, conference abstracts, or articles without full text.

### 2.3 Literature Coding and Quality Assessment

For the literature that met the criteria for meta‐analysis, each study was coded based on the following variables: (1) basic information (author and publication date), (2) type of experiment (between‐subjects or within‐subjects), (3) experiment location (field study or laboratory study), (4) altitude categories (1500–2500 m, 2500–4000 m, > 4000 m), (5) exposure time under high altitude (< 3 days, 3–7 days, 7–30 days, 30–365 days, > 365 days), (6) cognitive test used, and (7) sample size along with the mean and standard deviation for both sea‐level and high‐altitude experimental conditions. Furthermore, each test and sample were coded independently. If a study included multiple tests or conditions, they were coded separately allowing a single study to have multiple codes.

The Cochrane Risk of Bias Assessment Tool was used to assess the quality of the literature with Review Manager 5.3 software (Higgins and Green 2011). Seven entries were evaluated for bias risk across six areas: selection bias (including random sequence generation and allocation concealment), performance bias (blinding of participants and personnel), detection bias (blinding of outcome assessment), attrition bias (incomplete outcome data), reporting bias (selective reporting), and other bias. The bias risk for each of the seven entries was classified in the six categories as “low risk,” “high risk,” or “unclear” based on the established assessment criteria. Additionally, a bias risk chart was created to illustrate these evaluations.

### 2.4 Calculation of Effect Size

When analyzing continuous data, the standardized mean difference (SMD) is commonly used as both an outcome and a summary indicator for each study (Cohen 1988). The standard method for calculating SMD in a single trial is Cohen's *d*. However, in small sample studies, this simple calculation may introduce slight bias, leading to an overestimation of the effect size.

In contrast, Hedges' *g* is a similar metric that controls for this bias (Hedges 1981). Therefore, this study used Hedges' *g* as the effect size indicator to mitigate the risk of overestimating the effect size. Absolute values of Hedges' *g* of 0.2, 0.5, and 0.8 correspond to small, medium, and large effects, respectively (Cohen 1992). The effect size is negative if high‐altitude hypoxia exposure adversely affects cognitive function, and positive if it has no such effect.

### 2.5 Model Selection

The studies included in this meta‐analysis reported multiple effect sizes from the same sample, indicating that the effect sizes were not independent. In a traditional meta‐analysis, only one effect size is usually extracted per study (Assink and Wibbelink 2016), which fails to account for the correlations between effect sizes. This oversight can result in an overestimation of the overall effect size. This meta‐analysis employs a multilayered structure that can be expanded to better represent the data. The three‐level model considers the dependencies between effect sizes by incorporating an intermediate level, which captures variations within the same study. This approach enables the simultaneous inclusion of multiple effects from a single study (Cheung 2014). The three‐level meta‐analysis further decomposes the variance sources in effect sizes into three levels. Level 1 indicates errors stemming from the sampling method used in the original study. Level 2 represents the variance among multiple effect sizes within the same study, where significant results suggest heterogeneity. Level 3 refers to the variance in effect sizes across different studies, with significant results also indicating heterogeneity (Cheung 2014). In contrast to traditional meta‐analysis, a three‐level meta‐analysis effectively addresses dependencies between effect sizes from the same study, enhancing information retention and increasing statistical power (Assink and Wibbelink 2016). This study included various subgroups based on factors such as altitude and exposure time under high altitude, leading to the application of a three‐level meta‐analysis model to analyze the main effects and additional tests.

### 2.6 Heterogeneity Test and Moderated Effects Analysis

This study will use the one‐tailed log‐likelihood ratio tests (Assink and Wibbelink 2016; Lortie 2022). This method is primarily used to compare the goodness of fit between two probability distribution models with different parameters. Suppose the three‐level model outperforms the two‐level model (free estimation of Levels 1 and 2 or free estimation of Levels 1 and 3). In that case, it suggests significant between‐study or within‐study variance and heterogeneity in the main effects. This indicates the need for further moderated‐effects tests to identify the source of the heterogeneity (Assink and Wibbelink 2016; Cheung 2014; Van den Noortgate et al. 2013). In the moderated effects test, the moderating variables in this study—such as experimental design, type of experiment, altitude, high‐altitude exposure time, and cognitive test categorization—were added to the three‐level meta‐analytic model as covariates to estimate the moderated effect sizes (Assink and Wibbelink 2016; Cheung 2014).

### 2.7 Publication of Bias Testing and Sensitivity Analysis

Publication bias is the tendency for findings that are statistically significant to be published more frequently (Franco et al. 2014; Rodgers and Pustejovsky 2021). In this study, we will evaluate publication bias both qualitatively and quantitatively. This will be done using a funnel plot, Egger's MLMA regress, and the trim and fill method. A funnel plot helps determine if studies are evenly distributed on both sides of the total effect size; if they are, it suggests a low level of publication bias (Sterne and Harbord 2004). When the results of the Egger‐MLMA regression are not significant, it indicates a low level of publication bias (Rodgers and Pustejovsky 2021). If the Egger‐MLMA regression indicates significance or if the funnel plot displays an asymmetric distribution of effect sizes, the trim and fill method will be used to evaluate the impact of publication bias on the results of the meta‐analysis. If the effect sizes remain largely unchanged after clipping, it suggests that the influence of publication bias on the meta‐analysis is minimal (Duval and Tweedie 2000).

The stability of the meta‐analysis results will be assessed through sensitivity analysis, utilizing metrics such as outliers, the leave‐one‐out method for cross‐validation, and the safety factor approach. The statistical index for outlier analysis is the studentized deleted residual (SDR) (Viechtbauer and Cheung 2010). SDR measures the deviation between the observed size of a single effect and the average effect size. If the absolute value of SDR is more significant than 1.96, it indicates that the effect size is an outlier (Viechtbauer and Cheung 2010). The proportion of outliers should not exceed 1/10 of the total number of effect sizes (Viechtbauer and Cheung 2010). The leave‐one‐out method identifies studies that significantly affect the meta‐analysis results. It achieves this by systematically excluding each study and rerunning the meta‐analysis to determine if this exclusion alters the pooled effect sizes (Dodell‐Feder and Tamir 2018). Fail‐safe number assesses the reliability of results by determining how many additional negative outcomes would be required to invalidate the current conclusions when the meta‐analysis indicates significant findings (Rosenthal 1979). A higher fail‐safe number indicates greater stability in results. A fail‐safe number of 5*k* + 10 or more (where *k* is the number of original studies) suggests that the conclusions of the study are robust (Viechtbauer 2007).

### 2.8 Data Processing

In this study, a meta‐analysis was conducted using the metafor package for R version 4.3.1 (Viechtbauer 2010). Model parameters were estimated using the restricted maximum likelihood method (Viechtbauer 2005). A two‐tailed test with a *p*‐value less than 0.05 was considered significant.

## 3 Results

### 3.1 Literature Inclusion and Quality Assessment

After a thorough literature screening process, 59 papers published between 1966 and 2024 were included, resulting in a total of 739 effect sizes. The sample sizes in these studies ranged from 9 to 761 participants, and within each study, the effect sizes varied from 1 to 96. The quality of the literature was assessed using the Cochrane Risk of Bias Assessment Tool, as illustrated in Figures S1 and S2.

The studies examined a variety of cognitive test instruments to evaluate cognitive functioning in both plains and highlands subjects. A total of 60 papers analyzed 132 neuropsychological and behavioral tests, which were categorized into seven cognitive dimensions based on classification methods used in earlier reviews (H. Fan et al. 2024; Su et al. 2024; Yan 2014). These dimensions are executive control, working memory, long‐term memory, perceptual ability, psychomotor skill, attention, and various other undefined tests. See Table 1 for detailed cognitive test classification.

**TABLE 1:** Cognitive tasks and cognitive task categories.

Category	Cognitive test
Executive control	Auditory Response Control Caution Quotient, Flanker, Go/No‐Go, Masking, Majority Function Task, Mental Control Backward task, Stroop, Two‐Choice Oddball, Visual Response Control Caution Quotient, Visuospatial Executive Continuous Calculation, Serial Addition/Subtraction, Maze Test, Mathematical Processing, Mental Rotation Task, Raven's Standard Progressive Matrices, Simultaneous Spatial Processing, Trail Making Test, Mental Arithmetic Test
Working memory	Digit Span, WAIS‐RC Working Memory, Auditory Digit Span, Code Substitution Learning, Contralateral Delay Activity, Directional memory, Immediate Recall, Immediate Verbal Memory, Immediate Visual Memory, Memory Corridor, Memory Scanning, N‐Back, Serial Digit Learning Test, Spatial Memory Span, Visual Paired Association, Working Memory Spatial Task, Working Memory Verbal Task, Working Memory Visual Task, Digit Symbol Substitution, Meaningless Image Recognition, Mental Control Digit Serial Accumulation, Sternberg 6‐Letter Memory
Long‐term memory	Associative Learning, Figural Memory, Free Recall, Picture Recall Test, Picture Recognition Test, Visual Memory, Visual Reproduction, Visual Retention, WAIS‐RC‐Long Term Memory, Code Substitution Delayed Memory, Continuous Recognition, Continuous Recognition Memory, Delayed Recall, Delayed Verbal Memory, Delayed Visual Memory, Face recognition, Image Free Recall, Memory Recognition/Verbal Learning Task, Memory Search, Photo Memory, Picture Recognition, Portrait Feature Associative Recall, Procedural Memory, Remote Memory, Space Memory Test, Word‐Free Recall, Word Recognition
Perception	Visual 4‐Choice Reaction Time Test, Black‐Red Figure Test, Degraded Picture Naming, Displayed Letters Reading, Pattern Comparison, Visual Object Learning Task, Chroma Test, Color Brightness Discrimination, Critical Flicker Fusion Frequency, Length Discrimination, Match to Sample, Speed Estimate, Time Interval Perception, Bender Visual Motor Gestalt Test
Psychomotor skill	Benton Visual Retention Test, Audible Reaction Time, Auditory Recognition Reaction Time, Auditory Simple Reaction Time, Choice Reaction Time, Lighting Reaction Time, Procedural Reaction Time, Recognition Reaction Time, Simple Reaction Time, Visual Simple Reaction Time, Motor Praxis Test, Pursuit Aiming Test, Santa Anna, Finger Tapping Test, Psychomotor Vigilance Test, Simple Motor Tapping Test
Attention	Auditory Consistency Quotient, Auditory Endurance Quotient, Visual Consistency Quotient, Visual Endurance Quotient, Auditory Focus Quotient, Auditory Response Control Quotient, Auditory Speed Quotient, Full Scale Response Control Quotient, Number Search, Orientation Task, Visual Focus Quotient, Visual Response Control Quotient, Visual Search Goal Directed, Visual Search Stimulus Driven, Attention, Attention & Problem Solving, Attention Audible, Attention Distribution, Attention Network Task, Attention Span Test, Attention Switching Task, Auditory Attention Alert Quotient, Auditory Attention Distribution, Auditory Attention Quotient, Continuous Performance Test, Full Scale Attention Quotient, Spatial Attention Discrimination Task, Sustained attention, Visual Attention Alert Quotient, Visual Attention Distribution, Visual Attention Quotient, Visual Search Task
Others	Emotion Recognition, Language, Mind Body Coordination and Learning, Verbal Fluency Test‐Animals, Verbal Interference Test

### 3.2 Main Effects and Heterogeneity Tests

A three‐level meta‐analysis was conducted to estimate the main effect of the high‐altitude hypoxic environment on cognitive function. The overall mean effect size, based on Hedges' *g*, indicated a significant negative effect of the high‐altitude hypoxia on cognitive function (*g* = −0.424, SE = 0.105, 95% CI [−0.631, −0.217], *t* (737) = −4.017, *p* < 0.001).

The significance of within‐study (Level 2) and between‐study (Level 3) variances was analyzed using a one‐sided log‐likelihood ratio test, comparing a three‐level model (including both within‐study and between‐study variance) with a two‐level model (which included only one of these variances). The results showed significant differences in both within‐study variance (Level 2) (*σ* ^2^ = 0.57, *χ* ^2^ (1) = 6173.84, *p* < 0.001) and between‐study variance (Level 3) (*σ* ^2^ = 0.53, *χ* ^2^ (1) = 168.63, *p* < 0.001). Of the total sources of variance, sampling variance (Level 1) accounted for 1.96%, within‐study variance (Level 2) for 47.06%, and between‐study variance (Level 3) for 50.98%. These findings indicate significant heterogeneity in the main effects analyses, suggesting that further exploration of moderating variables is necessary to better understand the relationship between high‐altitude hypoxic environments and individual cognitive function.

### 3.3 Publication Bias and Sensitivity Testing

The funnel plot of the publication bias test is shown in Figure S3. The distribution of effect sizes in the plot is more dispersed and not symmetrically distributed around the overall effect size. The results of the Egger‐MLMA test (*β*_1_ = −7.976, *z* = −2.651, *p* < 0.05) suggest possible publication bias. Publication bias correction was conducted using the trim and fill method, where missing studies were manually added, and the symmetry position for each study was recalculated. After filling in 540 effect sizes, the result obtained was as follows: *g* = −0.444, SE = 0.107, 95% CI [−0.654, −0.234], *t* (737) = −4.415, *p* < 0.001. Cross‐validation results from the leave‐one‐out method showed a narrower interval of [−0.401, −0.389], with a predicted mean effect size (*g* = −0.424) close to the interval range. The safety coefficient was 576,371, much more significant than 5*k* + 10, suggesting that the meta‐analysis results are robust and less affected by publication bias.

The SDR outlier test identified 52 effect sizes with absolute values that differed from the mean effect size by more than 1.96, categorizing them as outliers. The proportion of outliers was less than 10% of the total effect sizes. The funnel plot after removing the outliers is shown in Figure S4. After remodeling, the mean effect size obtained was as follows: *g* = −0.340, SE = 0.059, 95% CI [−0.456, −0.225], *t* (685) = −5.775, *p* < 0.001. The Egger‐MLMA test showed that *β*_1_ = −74.4735, *z* = −13.443, *p* < 0.05, indicating that publication bias persisted. After manually filling in the missing studies and calculating the symmetric position of 507 effect sizes, the mean effect size after correction by the clipping method was as follows: *g* = −0.364, SE = 0.059, 95% CI [−0.479, −0.248], *t* (1192) = −6.188, *p* < 0.001. The effect sizes and confidence intervals before and after clipping were essentially unchanged. The leave‐one‐out method yielded [−0.357, −0.351], with a fail‐safe coefficient of 503,461, much larger than 5*k* + 10. Cross‐validation results from the leave‐one‐out method indicate that the meta‐analysis results are less affected by individual effect sizes, with the fail‐safe number all significantly larger than 5*k* + 10. Both methods suggest that the meta‐analysis results are reliable and less likely to be overturned.

### 3.4 Moderating Effects Test

Based on the significant differences between Levels 2 and 3, moderated effects analyses were conducted to account for variance and effect size heterogeneity. These analyses tested the effects of moderating variables on cognitive functioning in a high‐altitude hypoxic, with results presented in Table 2.

**TABLE 2:** Moderating effects of differences in cognitive ability between sea level and high altitude.

Moderator	*F* (df1, df2)	*k*	*β*	95% CIs		*t*	*F*	
				Lower	Upper		Level 2	Level3
Design	*F* (1, 685) = 0.157						0.618	0.383
Between‐study		321	−0.378	−0.739	−0.017	−2.053*		
Within‐study		418	−0.47	−0.75	−0.189	−3.29**		
Cognition	*F* (6, 680) = 3.427**						0.599	0.378
Executive control		161	−0.403	−0.668	−0.137	−2.975**		
Working memory		166	−0.322	−0.569	−0.074	−2.551*		
Long‐term memory		79	−0.572	−0.849	−0.295	−4.061***		
Perceptual		43	−0.923	−1.258	−0.587	−5.397***		
Psychomotor skill		128	−0.353	−0.609	−0.097	−2.707**		
Attention		144	−0.422	−0.686	−0.157	−3.131**		
Other		18	−0.362	−0.764	0.04	−1.768		
Exposure Time	*F* (4, 682) = 0.789						0.643	0.382
< 3 days		257	−0.527	−0.875	−0.18	−2.979**		
3 days		13	−0.067	−1.244	1.111	−0.111		
7–3 days		50	−0.265	−0.649	0.119	−1.355		
30–365 days		83	−0.379	−0.743	−0.016	−2.049*		
> 365 days		336	−0.437	−0.708	−0.165	−3.16**		
Altitude	*F* (2, 684) = 0.019						0.605	0.385
1500–2500m		32	−0.386	−0.946	0.174	−1.354		
2500–4000m		402	−0.439	−0.667	−0.212	−3.791***		
> 4000m		305	−0.436	−0.688	−0.184	−3.393***		

*Note*: *β*: average effect size in terms of Hedges' *g*; *k*: number of effect size; Level2: three‐level meta‐analysis Level 2; and Level3: three‐level meta‐analysis Level 3.

*p < 0.05, **p < 0.01, ***p < 0.001.

The moderating effect of the experimental design was not significant (*F* (1, 685) = 0.157, *p* = 0.692). Whether the researchers employed a within‐subjects design (*g* = −0.378, *p* = 0.04, 95% CI [−0.739, −0.017]) or a between‐subjects design (*g* = −0.470, *p* = 0.001, 95% CI [−0.750, −0.189]), both analyses indicated a significant negative impact of high‐altitude hypoxia on cognitive functioning.

The moderating effect of the cognitive test was significant (*F* (1, 685) = 3.427, *p* = 0.002). The high‐altitude hypoxic environment had the most significant adverse impact on long‐term memory (*g* = −0.572, *p* < 0.001, 95% CI [−0.849, −0.295]) and perceptual tasks (*g* = −0.923, *p* < 0.001, 95% CI [−1.258, −0.587]). This was followed by executive control (*g* = −0.403, *p* = 0.003, 95% CI [−0.668, −0.137]), psychomotor (*g* = −0.353, *p* = 0.07, 95% CI [−0.609, −0.097]), and attentional (*g* = −0.422, *p* = 0.002, 95% CI [−0.686, −0.157]). The final aspect is working memory (*g* = −0.322, *p* = 0.011, 95% CI [−0.569, −0.074]). No significant effects were observed on other cognitive classes (*g* = −0.362, *p* = 0.078, 95% CI [−0.764, −0.040]).

The moderating effect of exposure time was not significant (*F* (4, 682) = 0.789, *p* = 0.533). Analysis of the duration of high‐altitude hypoxic revealed a significant adverse impact on cognitive functioning for exposure time of less than 3 days (*g* = −0.527, *p* = 0.003, 95% CI [−0.875, −0.180]). Meanwhile, exposures lasting 3–7 days (*g* = −0.067, *p* = 0.912, 95% CI [−1.244, 1.111]) and 7–30 days (*g* = −0.265, *p* = 0.176, 95% CI [−0.649, −0.119]) did not significantly affect cognitive functioning. In contrast, exposures lasting 30–365 days (*g* = −0.379, *p* = 0.041, 95% CI [−0.743, −0.016]) and more prolonged than 365 days (*g* = −0.437, *p* = 0.002, 95% CI [−0.708, −0.165]) exhibited significant adverse effects on cognitive function.

The moderating effect of altitude was not significant (*F* (2, 684) = 0.019, *p* = 0.982). Comparisons among the three altitude categories revealed that hypoxic exposure at altitudes of 1500–2500 m had a nonsignificant effect on cognitive function (*g* = −0.386, *p* = 0.176, 95% CI [−0.946, −0.174]). However, at altitudes of 2500–4000 m (*g* = −0.439, *p* < 0.001, 95% CI [−0.667, −0.212]) and above 4000 m (*g* = −0.436, *p* < 0.001, 95% CI [−0.688, −0.184]), significant adverse effects of altitude hypoxia on cognitive function were observed.

**FIGURE 2:**
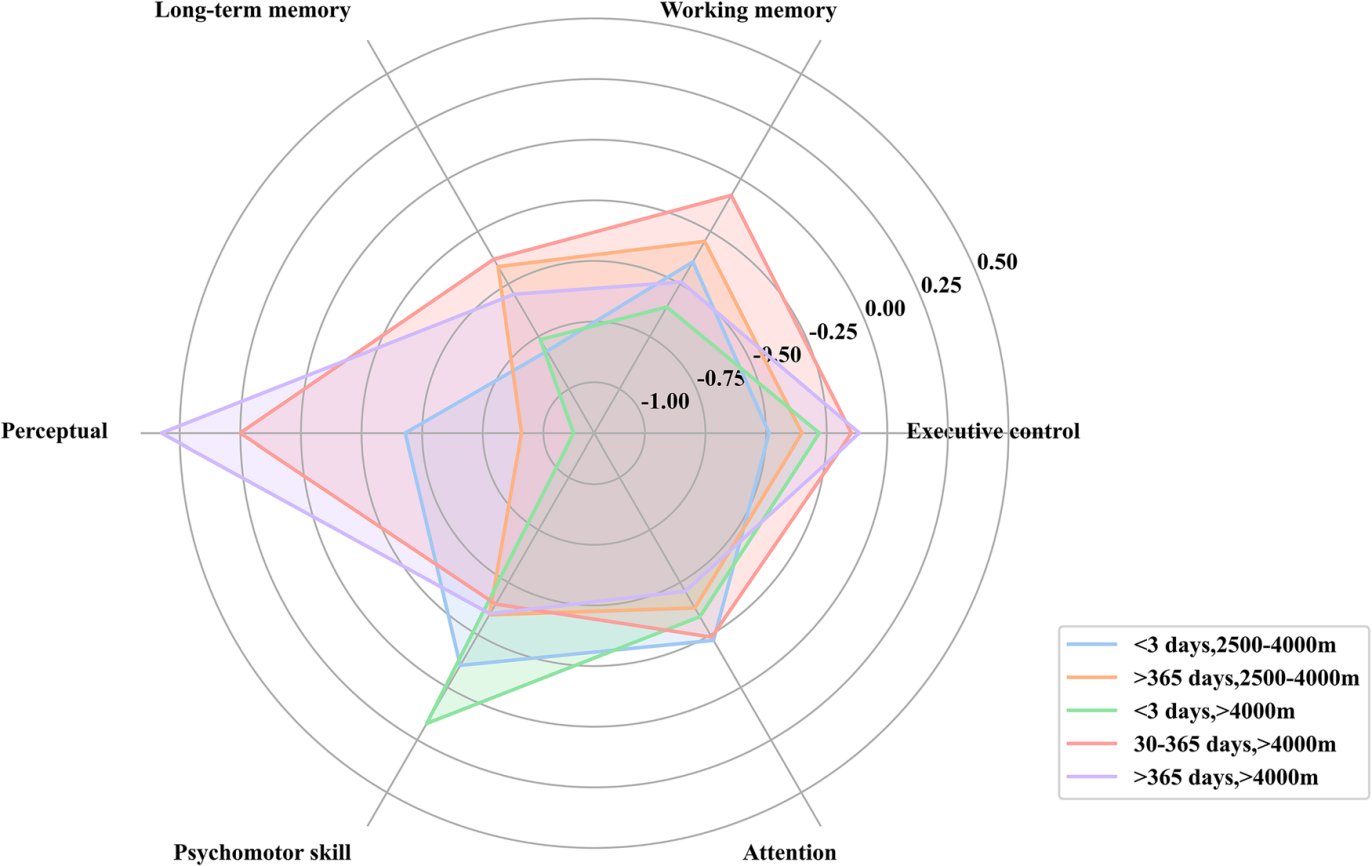
Subgroup analyzed radargrams showing only conditions for high‐altitude 2500–4000 m exposure < 3 days, 2500–4000 m exposure > 365 days, > 4000 m exposure < 3 days, > 4000 m exposure 30–365 days, and > 4000 m exposure >365 days.

Based on the categorization of exposure time, altitude, and cognitive function classification (excluding “other categories”), the overall mean effect sizes among the groups (5 × 3 × 6) were compared and assessed for heterogeneity. The results of the detailed subgroup analyses are presented in Table S1. Excluding null values, subgroups, including the following conditions: high‐altitude exposure of 2500–4000 m for < 3 days, 2500–4000 m for > 365 days, more significant than 4000 m for < 3 days, > 4000 m for 30–365 days, and > 4000 m for >365 days, provide the complete mean effect size across six cognitive dimensions: executive control, working memory, long‐term memory, perceptual, psychomotor skill, and attention. The results are illustrated in Figure 2.

## 4 Discussion

This study conducted a three‐level meta‐analysis to thoroughly evaluate the effects of different intensities of high‐altitude hypoxia exposure on cognitive function, as well as potential moderating variables. The results of the study were as follows: First, the main effect test in the three‐level meta‐analysis revealed significant negative impacts of high‐altitude hypoxia exposure on cognitive functioning, indicated by a moderately negative mean effect size.

Second, the test of moderating effects revealed that neither the high‐altitude exposure time nor the altitude itself had significant moderating impacts. However, cognitive performance showed a notable decline under conditions of acute high‐altitude exposure (< 3 days) and prolonged exposure (> 30 days). Importantly, cognitive performance also significantly decreased at altitudes above 2500 m. Additionally, the moderating effects of different cognitive test categories were significant. High‐altitude hypoxia had a more pronounced impact on long‐term memory and perceptual awareness. This was followed by effects on executive control, attention, psychomotor functioning, and finally, working memory.

Finally, the results of the meta‐analytic subgroup analyses revealed differences in cognitive function trends depending on the intensity of hypoxia exposure. Specifically, the sensitivity to hypoxia was greatest during acute exposure but decreased with longer acclimatization. Initially, acute hypoxia exposure had a limited impact on psychomotor skill; however, this effect became more pronounced with prolonged exposure and increased altitude. Additionally, memory demonstrated a stronger vulnerability to acute hypoxia exposure, which intensified at higher altitudes.

### 4.1 Effects of a High‐Altitude Hypoxic on Overall Cognitive Function

The main effects test in this three‐level meta‐analysis showed an overall mean effect of high‐altitude hypoxia on cognitive functioning of *g* = −0.424, indicating a significant negative impact on cognitive performance. Several studies support this conclusion (Li and Wang 2022; Wilson et al. 2009; Yan 2014). The exact mechanisms by which exposure to hypoxia affects brain function are not yet fully understood (Asmaro et al. 2013). However, it is known that these effects can occur through several pathways, including disruptions in cellular energy metabolism (Zheng et al. 2019), glutamate toxicity (Kushwah et al. 2018), impaired calcium overload (Socodato et al. 2018), and oxidative stress caused by free radicals (Pena et al. 2022). These mechanisms can damage neuronal, resulting in cognitive impairment and decreased performance in hypoxic environments.

### 4.2 Changes in Cognitive Function Across Exposure Intensities

Studies indicate that high‐altitude environments cause relative hypoxia in brain tissue, which inhibits sensory nerves and affects perception(Virues‐Ortega et al. 2006; Virues‐Ortega. et al. 2004). In this study, we discovered that the overall mean effect size of high‐altitude hypoxia on perception was *g* = −0.43 for exposures lasting < 3 days at altitudes between 2500 and 4000 m. This effect size increased to *g* = −1.12 at an altitude of 4000 m. This indicates that acute hypoxia has a more pronounced effect on perception, which intensifies with increasing altitude. These results align with previous findings (Dykiert et al. 2010; Virues‐Ortega. et al. 2004). Research shows that acute hypoxia in high altitude causes relative hypoxia in brain tissue, which inhibits perceptual nerves, lowers the perceptual threshold, and ultimately impairs the judgment of sounds at different intensities (Dykiert et al. 2010). During information processing, sensory information related to perception is transmitted from the sensory cortex to the prefrontal cortex (McMorris et al. 2017). This indicates that the recognition and processing of stimuli are associated with forebrain function. However, studies have found no significant structural or functional changes in the prefrontal cortex following prolonged exposure to high‐altitude hypoxia (X. Zhang and Zhang 2022). This lack of change may account for the absence of significant effects of long‐term hypoxic exposure on perceptual processes.

Psychomotor ability is the capacity to perceive, focus on, and respond to complex visual‐perceptual information while carrying out tasks that require fine motor coordination. The current study found that extended exposure to low oxygen levels significantly impaired psychomotor function, with the extent of impairment increasing at higher altitudes. Previous studies have similarly established a relationship between psychomotor function and altitude. Tune (1964) noted that psychomotor ability remains unaffected at altitudes below 3084 m but is progressively affected at elevations above this threshold (Luft 1961). For example, Kelman et al. (1969) found no decrease in psychomotor ability at a simulated altitude of 2438 m, likely due to the absence of detrimental effects at this lower altitude. However, Gedye (1964) reported a decrease in psychomotor functioning at the higher altitude of 3084 m. Psychomotor skill showed less sensitivity during the initial days of hypoxia exposure; however, their performance significantly declined with prolonged exposure, especially after more than 365 days. Previous studies have indicated that both motor speed and accuracy decrease in individuals experiencing extended high‐altitude hypoxia compared to those at lower altitudes (Berry et al. 1989; Hornbein et al. 1989; V. M. Sharma et al. 1975).

Neurophysiological studies suggest that the hippocampus and limbic system are vital for cognitive functions like learning and memory. These brain regions are also vulnerable to hypoxic conditions, which can impact the intensity and quality of neural activity (Terraneo and Samaja 2017). The impaired function of the hippocampus results in changes in the expression of synaptic proteins, such as synaptophysin and spiking protein, across various brain regions (R. Sharma et al. 2019). This alteration can significantly impact memory functions. The current study discovered that memory impairment caused by hypoxia worsens with increasing altitude, which aligns with findings reported by Yan (2014). Research shows that working memory experiences slight impairments at moderate altitudes of 2000–3000 m (Virues‐Ortega. et al. 2004). More significant declines are observed at altitudes between 3000 and 4000 m (Wilson et al. 2011), while substantial impairments in spatial memory occur above 5000 m (J. Zhang et al. 2011). At altitudes exceeding 6000 m, both encoding and short‐term memory are particularly affected (Bliemsrieder et al. 2022). It is suggested that hypoxia impacts memory during the encoding stage rather than during extraction (Wilson et al. 2009), with a more pronounced effect on storage compared to extraction, which is reversible(Virues‐Ortega. et al. 2004). However, this study found no improvement in memory function with extended exposure time, and prolonged hypoxic exposure adversely affected both working and long‐term memory. These findings suggest that the effects of hypoxia on memory warrant further research.

### 4.3 High‐Altitude Threshold and Acclimation

This study found that the high‐altitude hypoxic environment significantly impacts cognitive function only at altitudes above 2500 m. Although the common belief is that increasing altitude worsens the effects of hypoxia on cognitive abilities, this study revealed no significant difference in cognitive effects between altitudes of 3500 and 4000 m (*g* = −0.439) and those above 4000 m (*g* = −0.436). This may be due to the varying intensity of hypoxia's impact on different cognitive functions and the existence of an altitude threshold beyond which cognitive abilities are affected. For example, research suggests that an altitude of 2000 m is generally considered the lowest point at which the high‐altitude environment begins to impair essential cognitive functions such as attention and memory (J. Zhang et al. 2010). An altitude of 3500 m negatively affects daily activities such as work, study, and memory for adults, including children (Rimoldi et al. 2016). An altitude of 3860 m serves as the threshold where executive control is significantly affected during the conflict detection phase (Wei et al. 2021). However, this “altitude threshold” may vary depending on the experimental methods and tasks used by researchers. For instance, Merz et al. (2013) did not find a significant decrease in executive control functioning, as assessed by the Ruff Figural Fluency Test, at an altitude of 6265 m.

The study found no significant effect of hypoxic residence time on cognitive function in high altitude. Previous studies that used hypoxia acclimatization levels as indicators also failed to demonstrate significant moderating effects (Jung et al. 2020). The impact of exposure time on cognitive function is significant. This study found that acute hypoxic exposure notably affected cognitive abilities, resulting in decreased overall performance. However, the idea that longer exposure will lead to physiological acclimatization in individuals does not apply to cognitive function. Even after more than a year of living in high altitude area, cognitive performance remained significantly different compared to lowland controls. Increasing evidence indicates that there are persistent deficits in brain function upon returning to low altitude following brief exposure to hypoxia at high altitudes (Bonnon et al. 2000). Most current studies focus on high‐altitude habituation. To fully comprehend the effects of hypoxia on cognitive functioning in travelers, future research should include the entire process of acclimation and de‐acclimation.

### 4.4 Research Limitations

This study used a three‐level meta‐analysis to examine the combined effects of different intensities of hypoxia exposure on cognitive function. However, several shortcomings remain.

First, while the study aimed to be comprehensive and objective in its literature review, some subjectivity and bias may still exist. For example, weaknesses in the rigor and comprehensiveness of the inclusion and exclusion criteria could have led to the omission or incorrect exclusion of relevant studies, ultimately affecting the accuracy and reliability of the findings. Therefore, future research should strive to enhance the rigor and scientific basis of the literature screening criteria and methods.

Second, this study focused primarily on the direct effects of a high‐altitude hypoxic environment on cognitive function, but it did not take into account potential indirect effects. Individual factors such as intelligence, socioeconomic status, and conditions like sleep disorders and mood swings caused by hypoxia were overlooked. Therefore, future research should take a more comprehensive approach to assess the combined effects of the high‐altitude hypoxic environment on human cognitive function while also exploring relevant interventions and coping strategies.

Finally, this study offers a detailed and thorough categorization of cognitive tasks; however, it is important to note that this categorization is still subjective and lacks standardization. Furthermore, individual mental activities are complex and interconnected, which means that completing a specific task often requires the use of multiple cognitive abilities. Therefore, future research should aim to develop and implement standardized protocols for cognitive assessment.

## 5 Conclusion

This study thoroughly examined how different intensities of high‐altitude hypoxia exposure affect cognitive function and explored potential moderating variables using a three‐level meta‐analysis. The findings reveal that exposure to high‐altitude hypoxia significantly negatively impacts overall cognitive function, as indicated by a moderately negative mean effect size. However, the hypoxic environment at high altitude affects various cognitive functions to differing extents. Long‐term memory and perceptual functions are the most severely affected, followed by executive control, attention, psychomotor functions, and, lastly, working memory. The intensity of hypoxia exposure led to various changes in cognitive function, demonstrating significant alterations over time for both short‐term high‐altitude exposure (< 3 days) and long‐term exposure (> 30 days). Additionally, these changes were more pronounced at altitudes exceeding 2500 m.

## Author Contributions

**Yan Jiang**: investigation, writing–original draft. **Keer Cai**: data curation, writing–original draft. **Linling Zhu**: writing–review and editing. **Ming Fan**: writing–review and editing, resources. **Du‐Ming Wang**: resources, supervision, writing–review and editing. **Yong‐Qi Zhao**: resources, supervision, writing–review and editing.

## Conflicts of Interest

The authors declare no conflicts of interest.

## Peer Review

The peer review history for this article is available at https://publons.com/publon/10.1002/brb3.70883

## Supporting information




**Supplementary Materials**: brb370883‐sup‐0001‐SuppMat.docx

## Data Availability

The raw data supporting the conclusions of this article will be made available by the authors without undue reservation.
