# Supplementary material for: A Meta‐Analysis of Multidimensional Cognitive Functions Changes in Different Intensities of High‐Altitude Hypoxia
Source: Brain Behav. 2025 Sep 21;15(9):e70883. doi: 10.1002/brb3.70883 (PMC12451029; doi:10.1002/brb3.70883)
Supplement: Supplementary file 1 — Supplementary Materials: brb370883‐sup‐0001‐SuppMat.docx [file BRB3-15-e70883-s001.docx]

Figure S1：Risk of bias graph


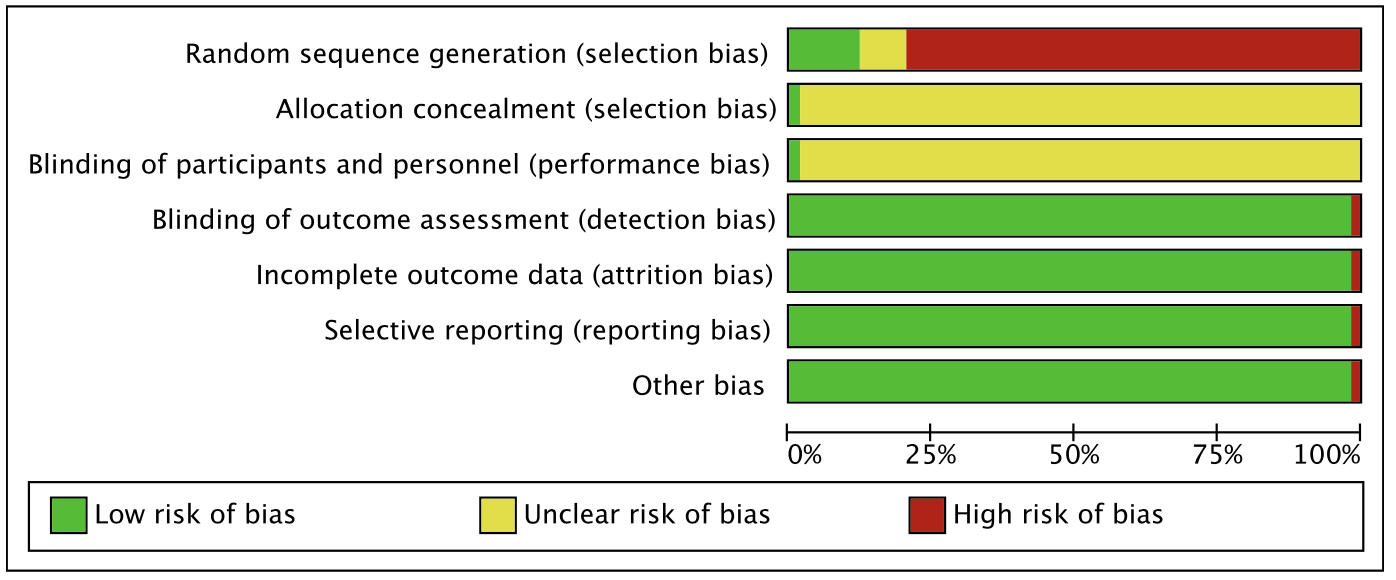


Figure S2：Risk of bias summary


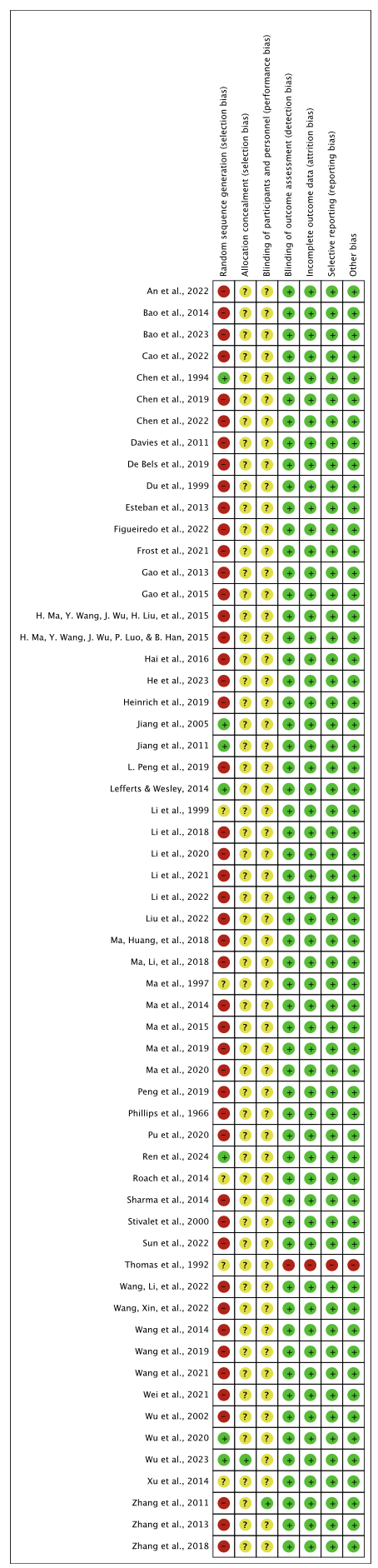


Figure S3：Funnel Plot-739 effect size.


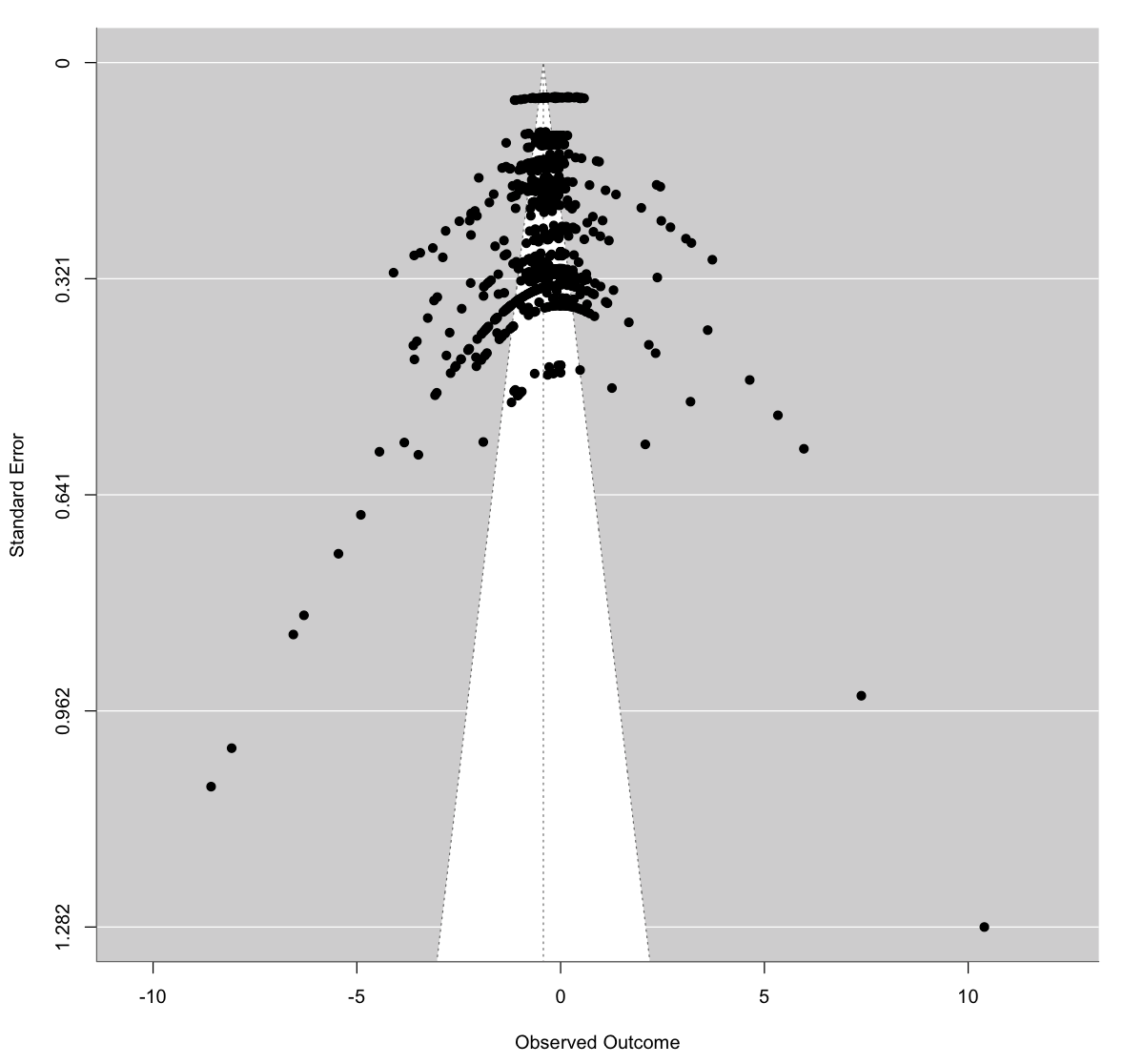


Figure S4：Funnel plot-687 effect size after excluding the outliers.


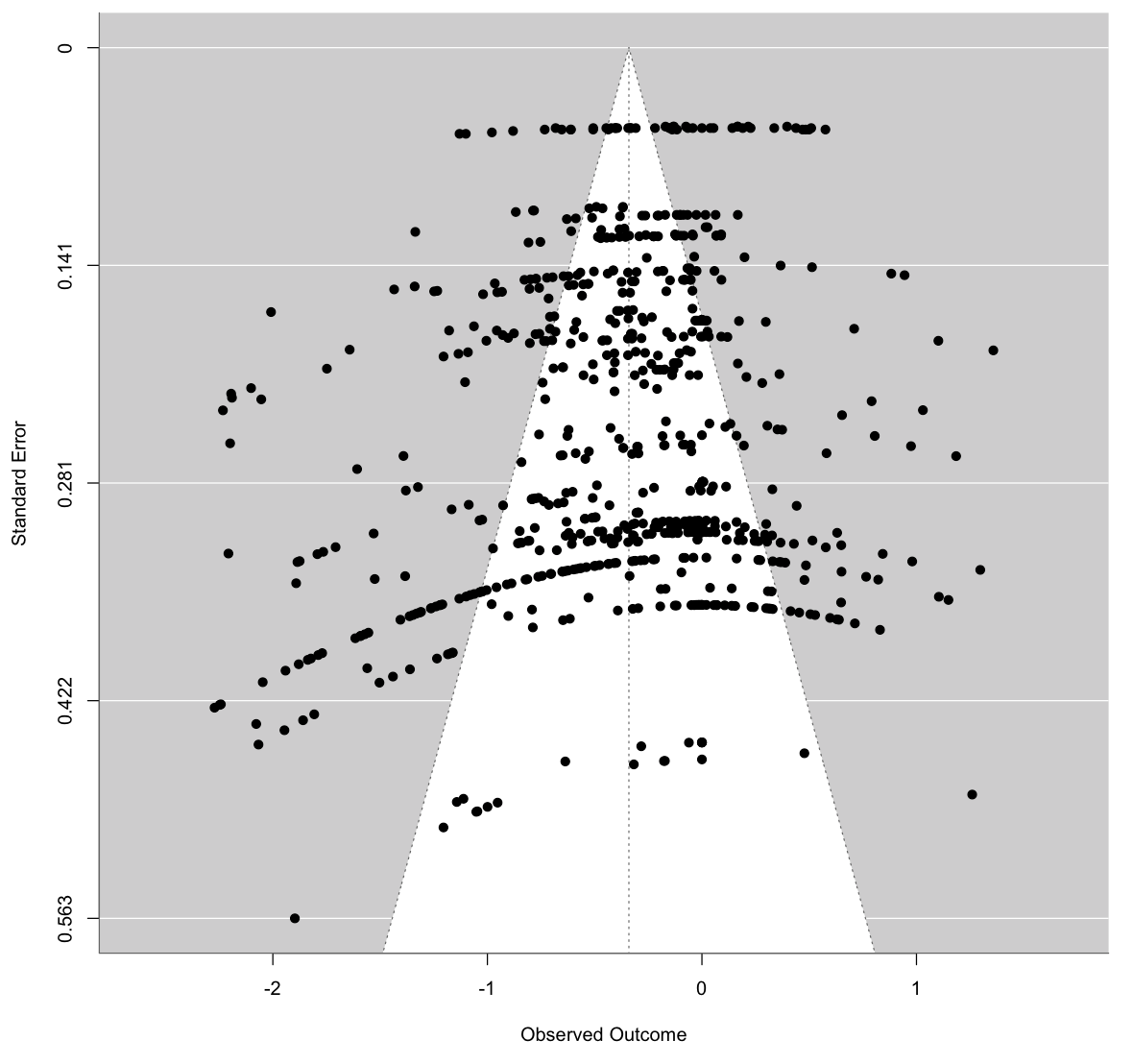


Table S1：The results of the subgroup analysis were grouped according to the type of cognitive test, altitude, and exposure time at high altitude

| **Tasktype** | **Altitude(m)** | **Time(day)** | **β** | **t** | **95% CIs** | |
| --- | --- | --- | --- | --- | --- | --- |
|  |  |  |  |  | **Lower** | **Upper** |
| Executive Control | 1500-2500 | 30-365 | -0.31 | -0.64 | -1.26 | 0.64 |
|  | 2500-4000 | <3 | -0.49 | -2.00 * | -0.97 | -0.01 |
|  |  | >365 | -0.35 | -3.97 *** | -0.53 | -0.18 |
|  | >4000 | <3 | -0.28 | -2.27 * | -0.52 | -0.04 |
|  |  | 7-30 | -0.09 | -0.22 | -0.84 | 0.67 |
|  |  | 30-365 | -0.15 | -1.23 | -0.38 | 0.09 |
|  |  | >365 | -0.12 | -1.18 | -0.31 | 0.08 |
| Working Memory | 1500-2500 | 30-365 | -0.58 | -3.62 *** | -0.89 | -0.26 |
|  |  | >365 | -0.28 | -1.60 | -0.63 | 0.06 |
|  | 2500-4000 | <3 | -0.40 | -4.33 *** | -0.57 | -0.22 |
|  |  | 3-7 | 0.05 | 0.21 | -0.43 | 0.53 |
|  |  | 7-30 | -0.84 | -3.51 *** | -1.31 | -0.37 |
|  |  | 30-365 | -0.95 | -5.25 *** | -1.31 | -0.60 |
|  |  | >365 | -0.30 | -3.87 *** | -0.45 | -0.15 |
|  | >4000 | <3 | -0.61 | -4.30 *** | -0.89 | -0.33 |
|  |  | 30-365 | -0.08 | -0.46 | -0.41 | 0.26 |
|  |  | >365 | -0.49 | -4.62 *** | -0.70 | -0.28 |
| Long-term Memory | 1500-2500 | 30-365 | -0.03 | -0.16 | -0.39 | 0.33 |
|  |  | >365 | -0.78 | -1.72 | -1.67 | 0.11 |
|  | 2500-4000 | <3 | -0.82 | -6.81 *** | -1.06 | -0.58 |
|  |  | 7-30 | -0.80 | -1.66 | -1.75 | 0.14 |
|  |  | 30-365 | -0.51 | -1.04 | -1.46 | 0.45 |
|  |  | >365 | -0.42 | -2.31 * | -0.77 | -0.06 |
|  | >4000 | <3 | -0.77 | -5.26 *** | -1.05 | -0.48 |
|  |  | 30-365 | -0.38 | -2.10 * | -0.74 | -0.03 |
|  |  | >365 | -0.55 | -4.24 *** | -0.80 | -0.30 |
| Perception | 1500-2500 | 30-365 | -0.14 | -0.28 | -1.10 | 0.82 |
|  | 2500-4000 | <3 | 12 | -3.17 ** | -0.70 | -0.16 |
|  |  | >365 | -0.91 | -3.39 *** | -1.44 | -0.38 |
|  | >4000 | <3 | -1.12 | -4.66 *** | -1.60 | -0.65 |
|  |  | 30-365 | 0.25 | 0.94 | -0.27 | 0.77 |
|  |  | >365 | 0.58 | 1.29 | -0.30 | 1.45 |
| Psychomotor skill | 2500-4000 | <3 | -0.10 | -1.14 | -0.28 | 0.08 |
|  |  | 3-7 | -0.24 | -1.44 | -0.56 | 0.09 |
|  |  | 7-30 | -0.73 | -4.05 *** | -1.09 | -0.38 |
|  |  | 30-365 | -0.85 | -3.50 *** | -1.33 | -0.38 |
|  |  | >365 | -0.35 | -3.14 ** | -0.56 | -0.13 |
|  | >4000 | <3 | 0.17 | 0.84 | -0.23 | 0.57 |
|  |  | 7-30 | 0.13 | 0.32 | -0.66 | 0.93 |
|  |  | 30-365 | -0.40 | -2.46 * | -0.71 | -0.08 |
|  |  | >365 | -0.35 | -3.96 *** | -0.52 | -0.18 |
| Attention | 1500-2500 | >365 | -0.61 | -2.31 * | -1.12 | -0.09 |
|  | 2500-4000 | <3 | -0.22 | -1.75 | -0.48 | 0.03 |
|  |  | 7-30 | -0.54 | -1.63 | -1.19 | 0.11 |
|  |  | >365 | -0.38 | -4.66 *** | -0.54 | -0.22 |
|  | >4000 | <3 | -0.34 | -3.26 ** | -0.54 | -0.13 |
|  |  | 7-30 | -0.44 | -4.32 *** | -0.64 | -0.24 |
|  |  | 30-365 | -0.24 | -1.07 | -0.68 | 0.20 |
|  |  | >365 | -0.46 | -3.94 *** | -0.69 | -0.23 |
| Note：Tasktype: cognitive task categories；β: average effect size in terms of Hedge's g  *p <0.05, **p <0.01, ***p <0.001. | | | | | | |
